# Supplementary material for: Simplicilones A and B Isolated from the Endophytic Fungus Simplicillium subtropicum SPC3
Source: Antibiotics (Basel). 2020 Oct 29;9(11):753. doi: 10.3390/antibiotics9110753 (PMC7693999; doi:10.3390/antibiotics9110753)
Supplement: Supplementary file 1 [file antibiotics-09-00753-s001.pdf]

Supplementary Materials

Simplicilones A and B isolated from the endophytic fungus *Simplicillium subtropicum* SPC3

Elodie Gisele M. Anoumedem [1], Bel Youssouf G. Mountessou [1], Simeon F. Kouam [1,*], Abolfazl Narmani [2,3] and Frank Surup [2,4,*]

[1] Department of Chemistry, Higher Teacher Training College, University of Yaoundé I, P.O. Box 47, Yaoundé, Cameroon; kfogue@yahoo.com; gisele.elodie@yahoo.com (EGMA); mountessou@yahoo.com (BYGM)
[2] Microbial Drugs Department, Helmholtz-Centre for Infection Research (HZI), Inhoffenstr. 7, 38124 Braunschweig, Germany; frank.surup@helmholtz-hzi.de (FS)
[3] Department of Plant Protection, Faculty of Agriculture, University of Tabriz, Tabriz, Iran
[4] German Centre for Infection Research (DZIF), partner site Hannover-Braunschweig, 38124 Braunschweig, Germany

* Correspondence: marc.stadler@helmholtz-hzi.de (M.S.); kfogue@yahoo.com (S.F.K.)

Contents

Molecular phylogenetic analysis

Table S1: Results of the megablast search analysis at NCBI's GenBank nucleotide database for strain SPC3

Figure S1: Consensus phylogram (75% majority rule) of 1002 trees resulting from a Bayesian analysis of ITS sequence alignment using MrBayes v. 3.2.2 of various *Simplicilium* species. The scale bar indicates 0.04 expected changes per site. The tree was rooted to *Verticillium fungicola var. fungicola* CBS 992.69 (EF641889.1)

Figure S2: HRESI-Mass spectrum of simplicilone A (**1**)

Figure S3: ^{1}H NMR (500 MHz, $CDCl_3$) spectrum of simplicilone A (**1**)

Figure S4: ^{13}C NMR (500 MHz, $CDCl_3$) spectrum of simplicilone A (**1**)

Figure S5: HSQC spectrum of simplicilone A (**1**)

Figure S6: HMBC spectrum of simplicilone A (**1**)

Figure S7: ROESY spectrum of simplicilone A (**1**)

Figure S8: HRESI-Mass spectrum of simplicilone B (**2**)

Figure S9: ^{1}H NMR (500 MHz, $CDCl_3$) spectrum of simplicilone B (**2**)

Figure S10: ^{13}C NMR (500 MHz, $CDCl_3$) spectrum of simplicilone B (**2**)

Figure S11: HSQC spectrum of simplicilone B (**2**)

Figure S12: HMBC spectrum of simplicilone B (**2**)

Figure S13: COSY spectrum of simplicilone B (**2**)

Figure S14: ROESY spectrum of simplicilone B (**2**)

Molecular phylogenetic analysis

Megablast search analysis at NCBI's GenBank nucleotide database showed high similarity with reference sequence of *Simplicilium* species from GenBank (Table S1). A phylogeny inferred based on ITS-rDNA sequence obtained in this study together with sequence data from GenBank. The final sequence alignment of the ITS-rDNA sequence comprising 20 internal taxa had 612 characters and 140 unique site patterns. Verticillium fungicola var. fungicola CBS 992.69 (EF641889.1) served as the outgroup taxon. Bayesian analyses were performed using the best-fitting substitution model (K80+I+G) and resulted in 1002 generations. After discarding the first 25% of generations as burn-in, the remaining 752 (75%) generations were used to calculate the consensus Bayesian tree and posterior probabilities. Results indicated that the isolate used in this study clustered with *Simplicillium subtropicum* (Figure S1).

Table S1: Results of the megablast search analysis at NCBI's GenBank nucleotide database for strain SPC3.

Description	Max Score	Total Score	Query Cover	E value	Per. Ident	Accession
Simplicillium sp. strain DTO 391-A3	1123	1123	100%	0	99.51%	MN788113.1
Simplicillium sp. H198	1123	1123	100%	0	99.51%	KP184324.1
Simplicillium sp. H41	1118	1118	99%	0	99.51%	KT318874.1
Simplicillium sp. H133	1105	1105	98%	0	99.51%	KP184319.1
Simplicillium sp. H174	1103	1103	98%	0	99.51%	KP184323.1
Simplicillium sp. strain H261	1099	1099	97%	0	99.50%	KX020567.1
Simplicillium sp. strain H168	1099	1099	97%	0	99.50%	KX020563.1
Simplicillium sp. H61	1099	1099	97%	0	99.50%	KP184314.1
Simplicillium sp. H161	1096	1096	97%	0	99.50%	KP184321.1
Simplicillium sp. H63	1096	1096	97%	0	99.50%	KP184316.1
Simplicillium sp.	1086	1086	96%	0	99.50%	MT635320.1
Simplicillium sp. H44	1051	1051	93%	0	99.48%	KP184312.1
Ascomycota sp. isolate F-20	1029	1029	91%	0	99.47%	MT610994.1
Simplicillium sp. strain MF44	990	990	88%	0	99.45%	KY397989.1
Simplicillium sp. M-27	1074	1074	95%	0	99.33%	JQ422627.1
Simplicillium sp. H36	1053	1053	94%	0	99.31%	KP184311.1
Simplicillium sp. JCM 39053	1033	1033	92%	0	99.30%	LC496873.1
Simplicillium sp. JCM 39052	1033	1033	92%	0	99.30%	LC496872.1
Uncultured *Simplicillium* clone DZ_69	1026	1026	91%	0	99.30%	KX214368.1
Uncultured *Simplicillium* clone DZ_62	1026	1026	91%	0	99.30%	KX214365.1
Simplicillium lanosoniveum strain SlB15C01	994	994	89%	0	99.27%	MK952446.1
Simplicillium lanosoniveum strain SilGD65	994	994	89%	0	99.27%	MH483712.1
Simplicillium sp. strain SZWL8	1042	1042	94%	0	99.14%	MH186014.1
Simplicillium sp. isolate SCSIO41513	1027	1027	92%	0	99.13%	MT436779.1
Simplicillium subtropicum strain SsB17A02	1003	1003	90%	0	99.11%	MK952451.

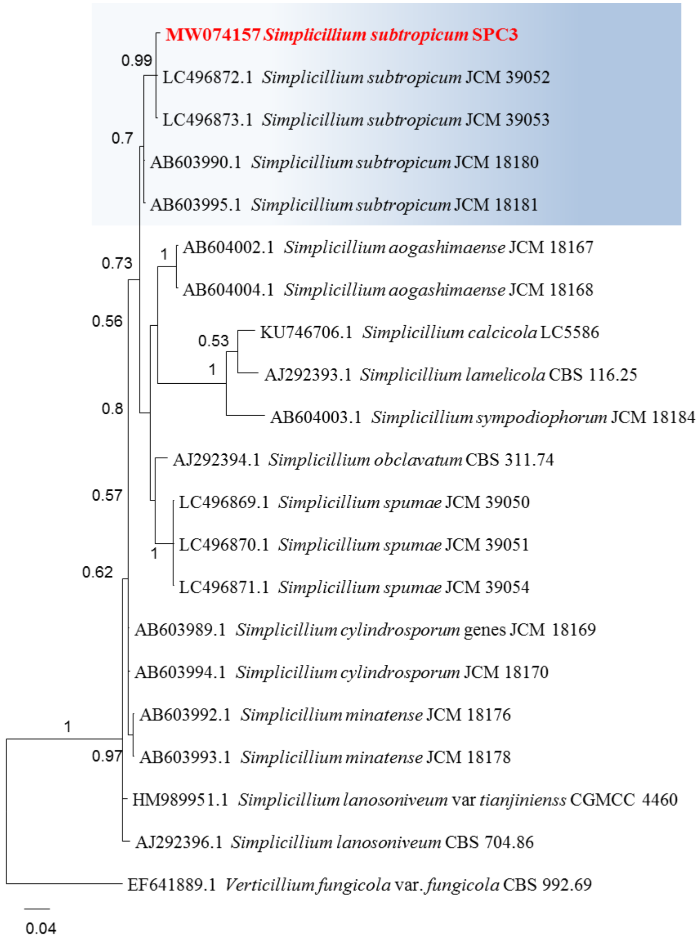

Figure S1: Consensus phylogram (75% majority rule) of 1002 trees resulting from a Bayesian analysis of ITS sequence alignment using MrBayes v. 3.2.2 of various *Simplicilium* species. The scale bar indicates 0.04 expected changes per site. The tree was rooted to *Verticillium fungicola var. fungicola* CBS 992.69 (EF641889.1).

Table S2: Minimum inhibitory concentrations (MIC) of **1**–**2**. n.i.: no inhibition up to 66.7 µg/mL

**test organism**	**strain number**	**MIC [µg×mL^{-1}]**	
		**1**	**2**
organism			
Bacillus subtilis	DSM 10	n.i.	n.i.
Staphylococcus aureus	DSM 346	n.i.	n.i.
Micrococcus luteus	DSM 1790	n.i.	n.i.
Chromobacterium violaceum	DSM 30191	n.i.	n.i.
Escherichia coli	DSM 1116	n.i.	n.i.
Pseudomonas aeruginosa	PA14	n.i.	n.i.
Mycolicibacterium smegmatis	ATCC 700084	n.i.	n.i.
Candida albicans	DSM 1665	n.i.	n.i.
Schizosaccharomyces pombe	DSM70572	n.i.	n.i.
Mucor hiemalis	DSM 2656	n.i.	n.i.
Pichia anomala	DSM 6766	n.i.	n.i.
Rhodotorula glutinis	DSM 10134	n.i.	n.i.

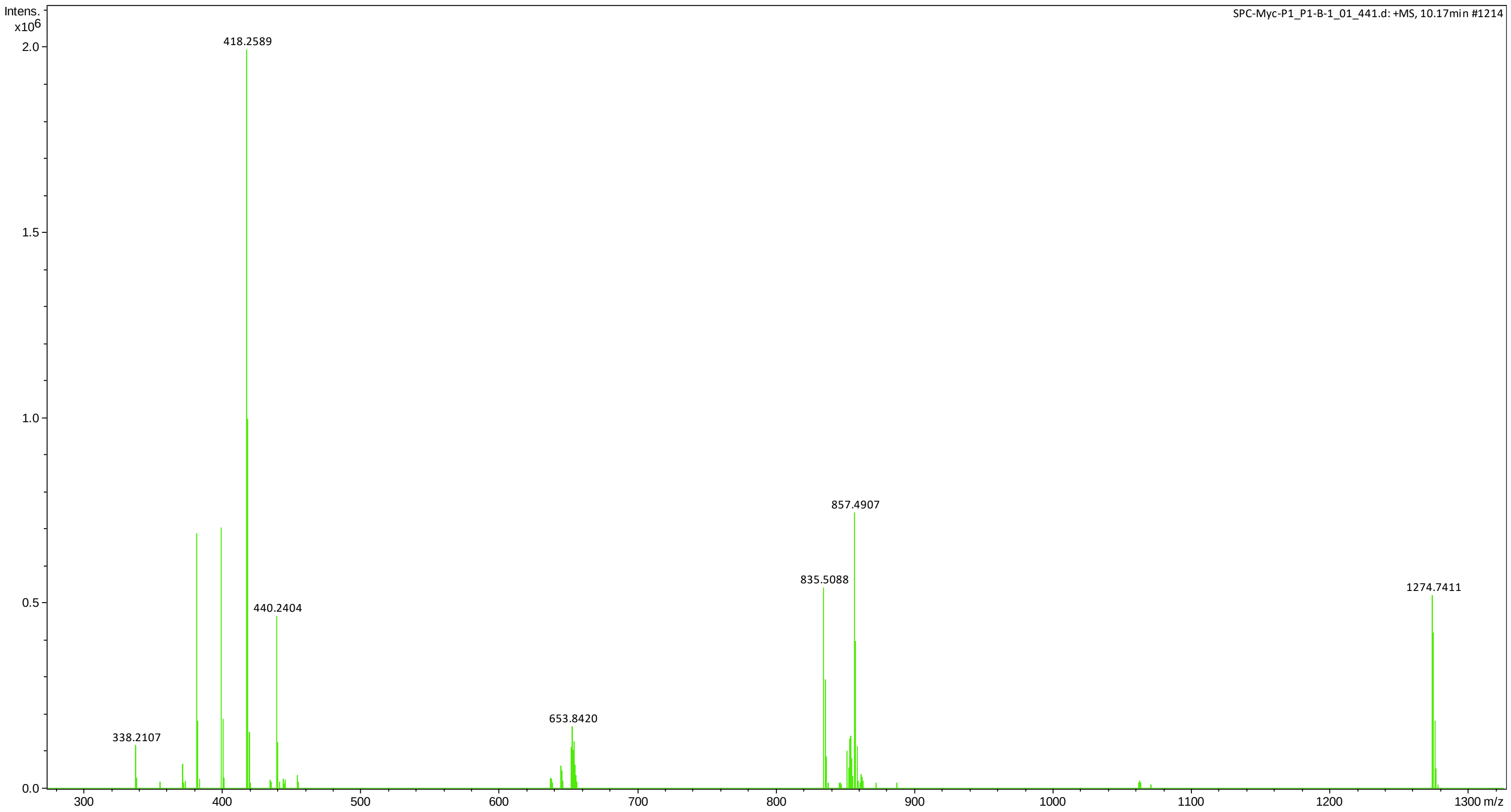

Figure S2: HRESI-Mass spectrum of simplicilone A (**1**)

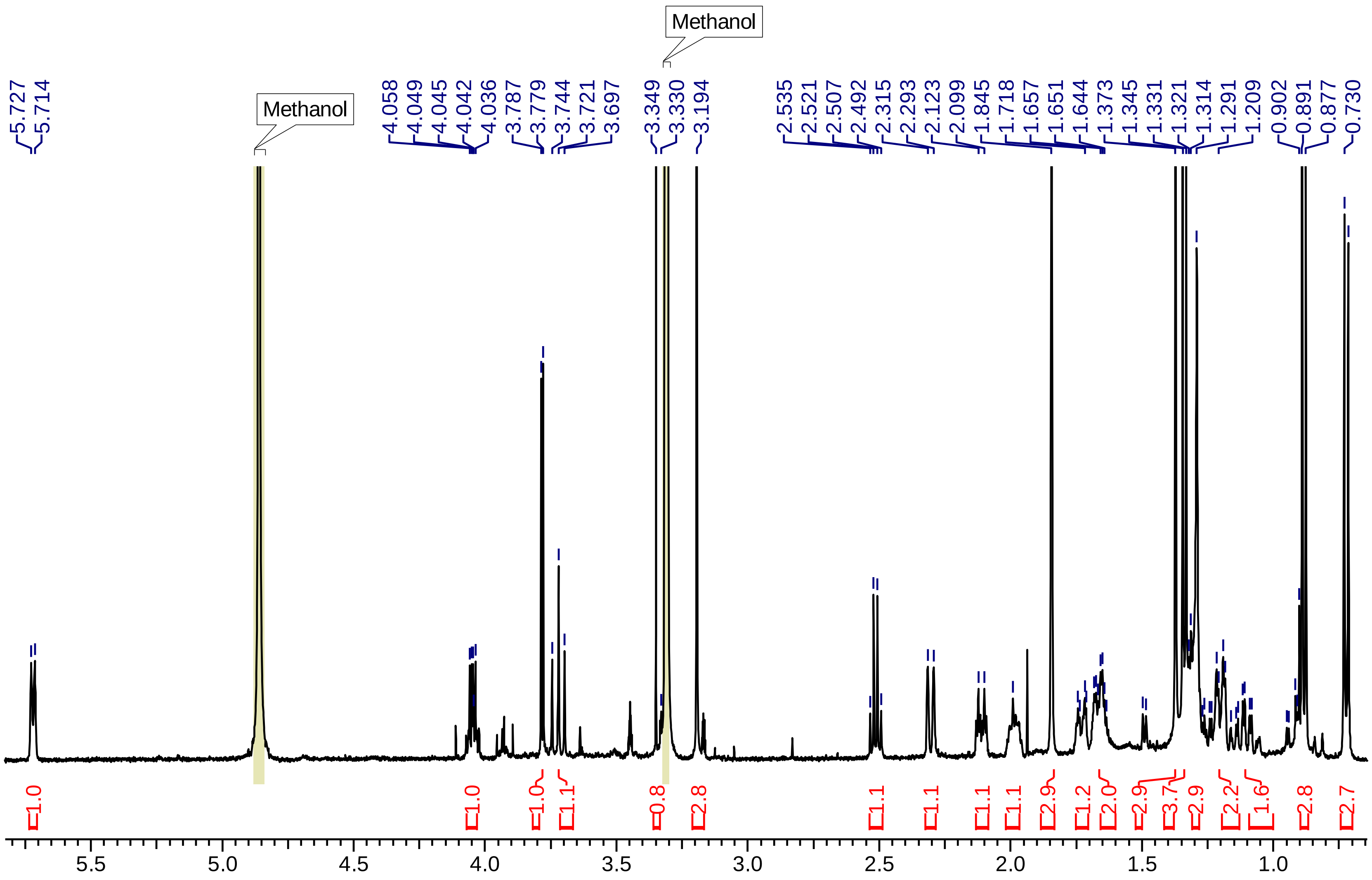

Figure S3: ^{1}H NMR (500 MHz, $CDCl_3$) spectrum of simplicilone A (**1**)

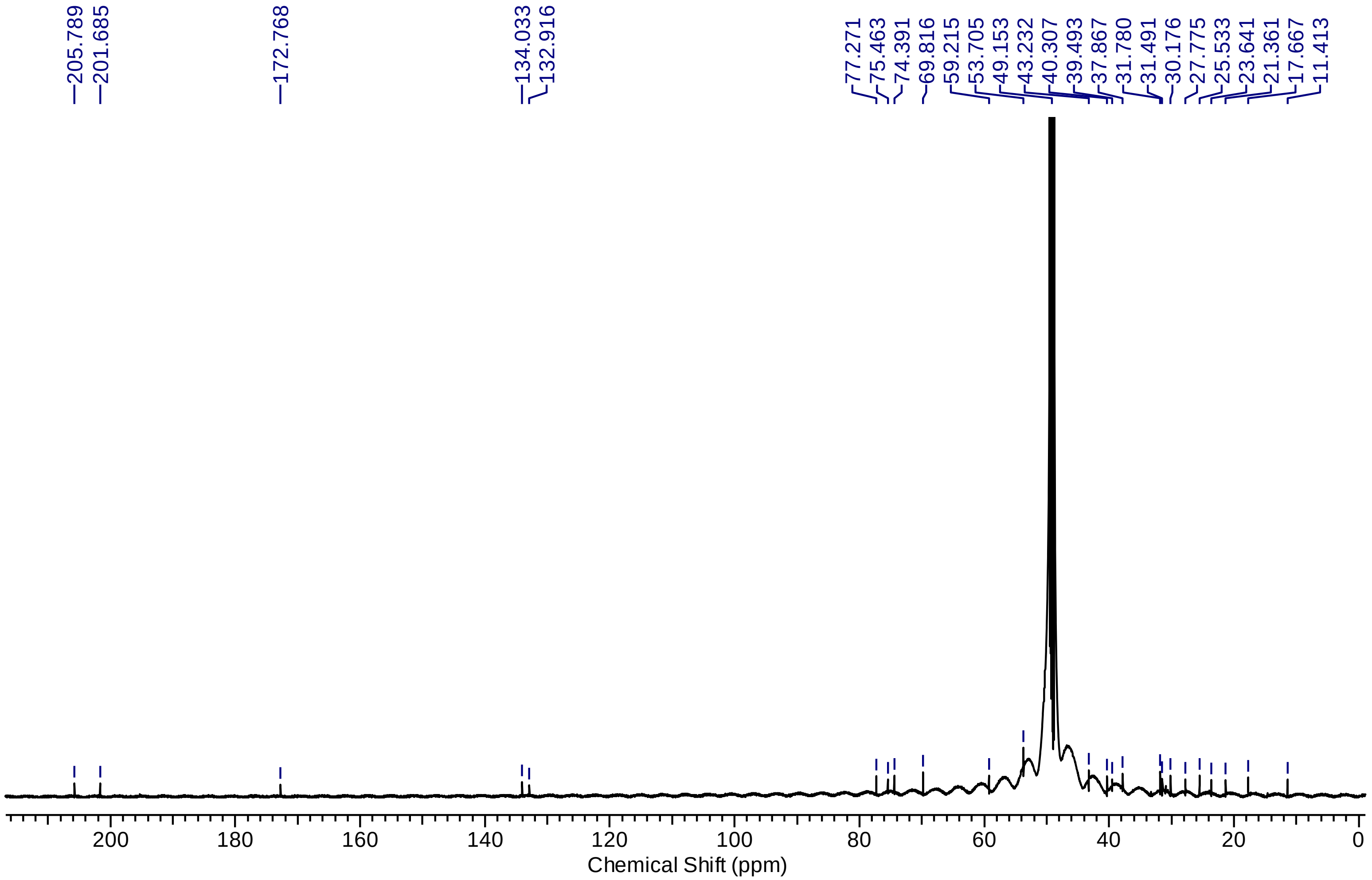

Figure S4: ^{13}C NMR (500 MHz, $CDCl_3$) spectrum of simplicilone A (**1**)

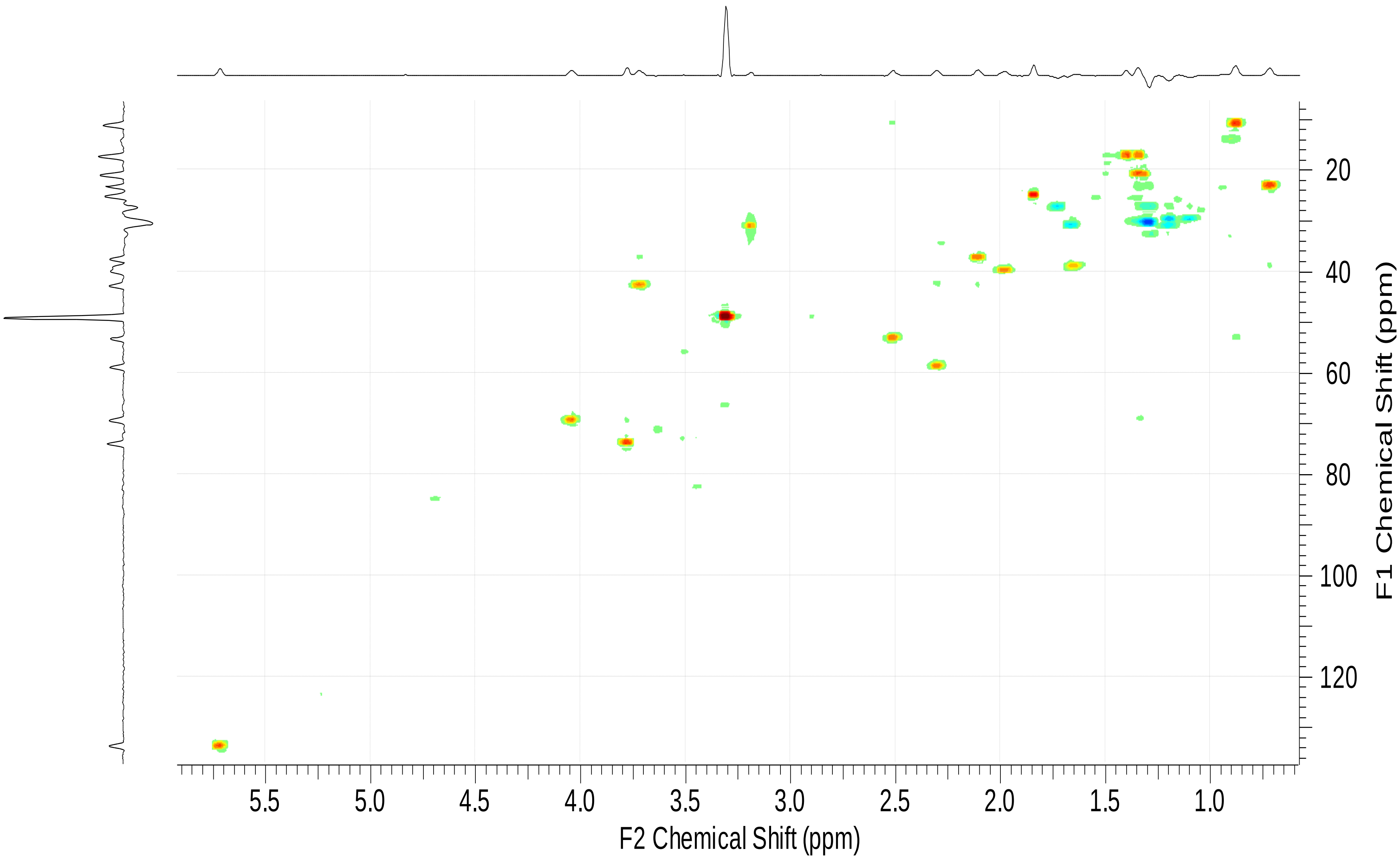

Figure S5: HSQC spectrum of simplicilone A (**1**)

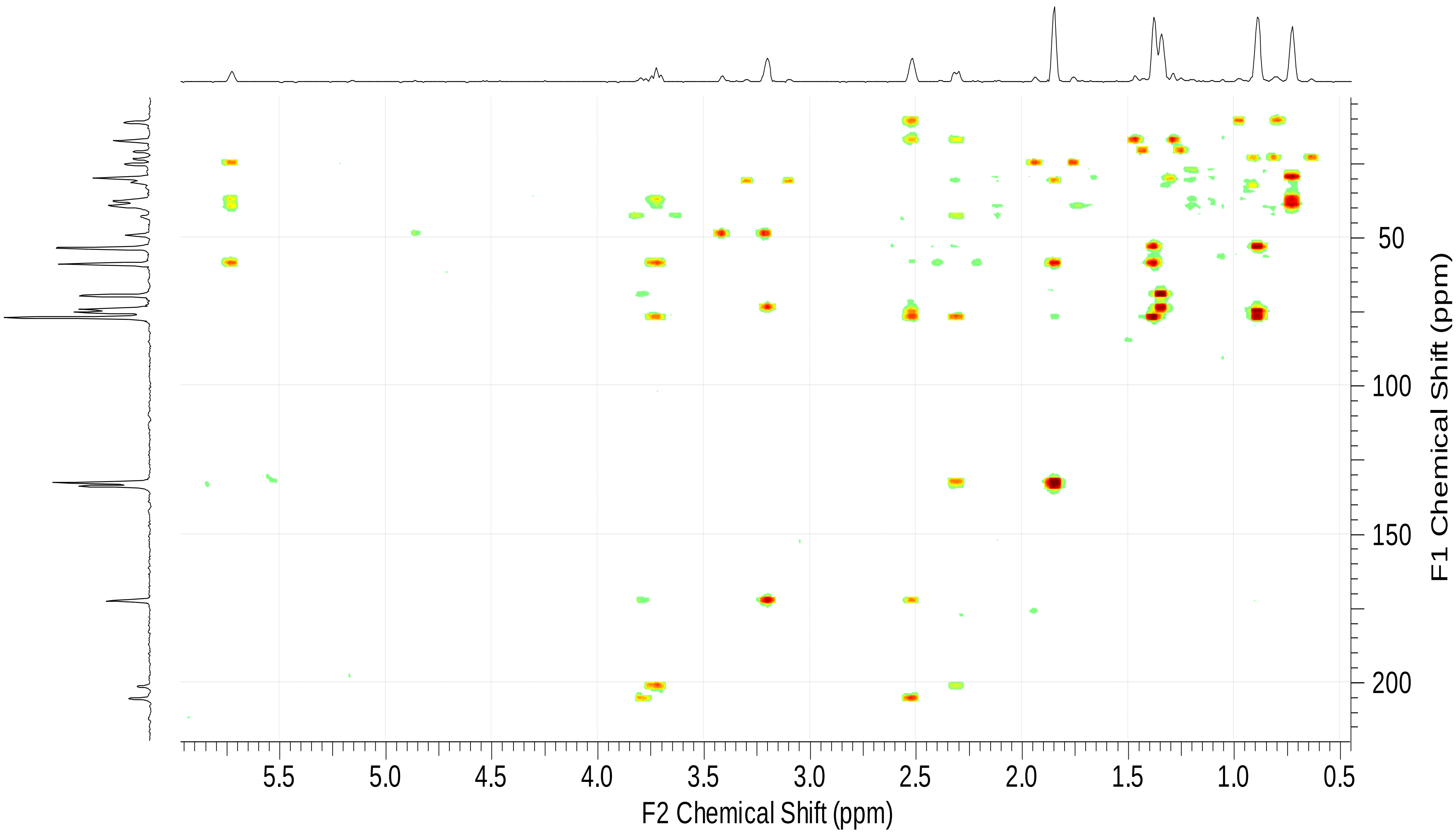

Figure S6: HMBC spectrum of simplicilone A (**1**)

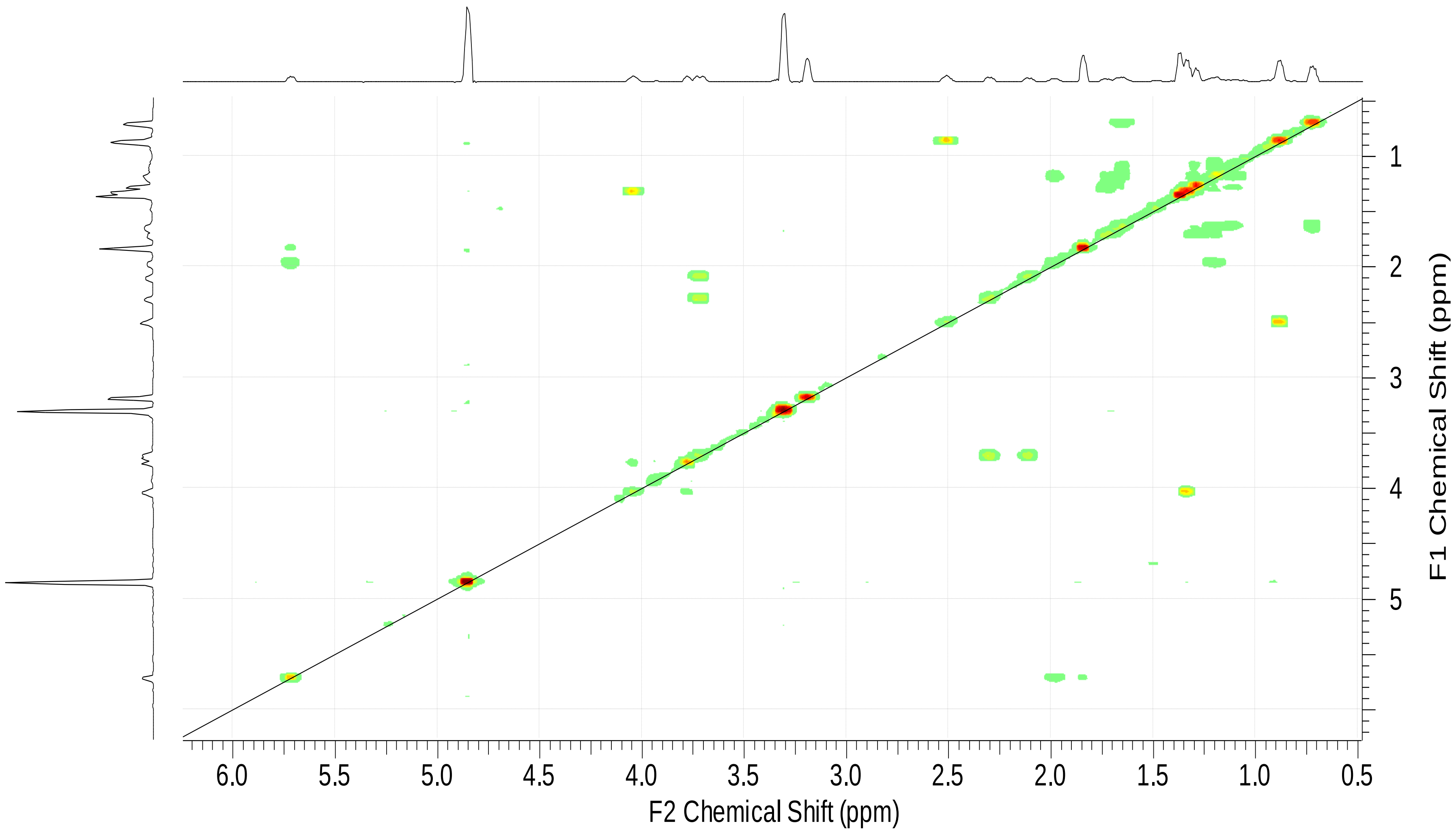

Figure S7: ROESY spectrum of simplicilone A (**1**)

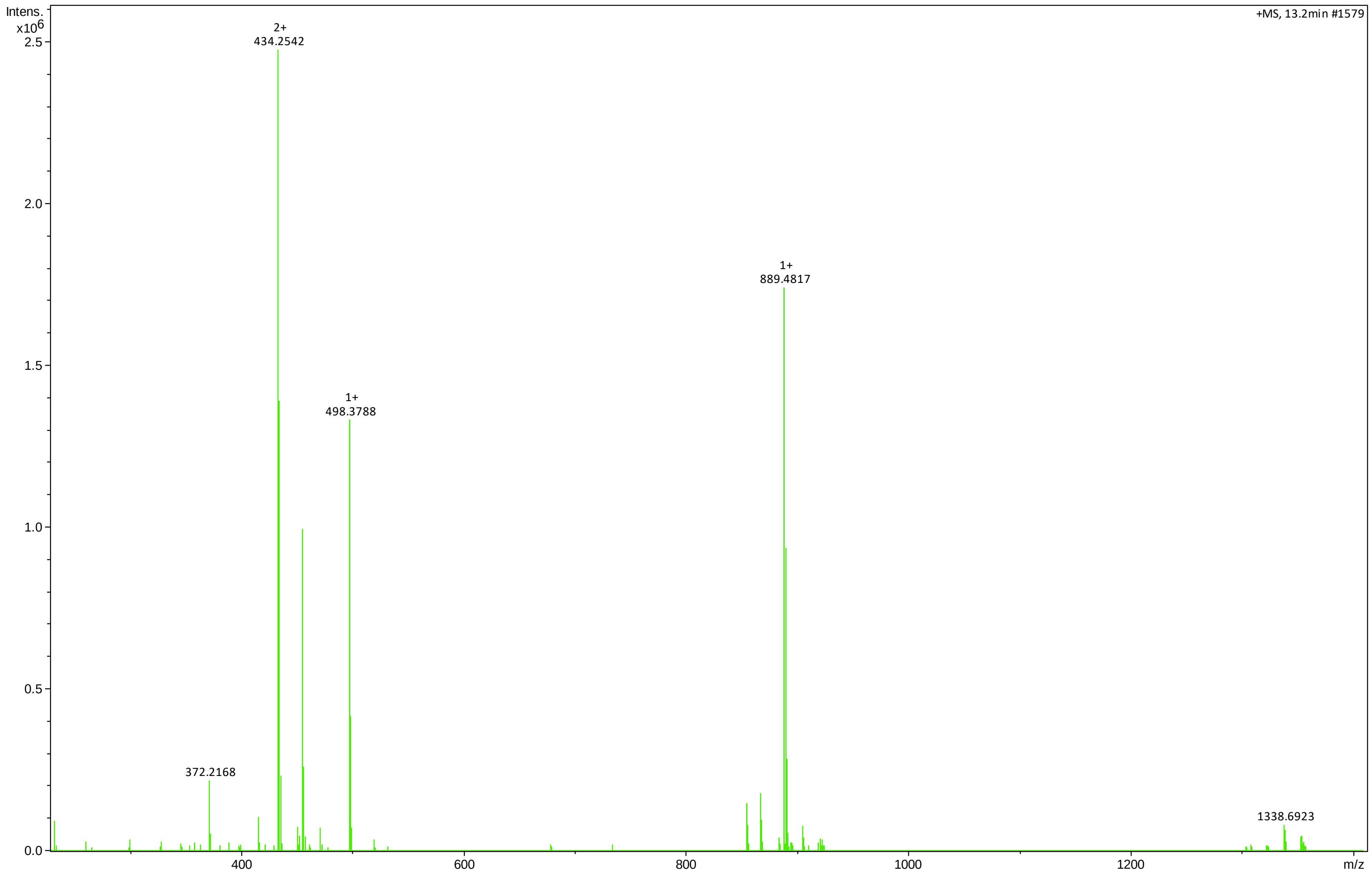

Figure S8: HRESI-Mass spectrum of simplicilone B (**2**)

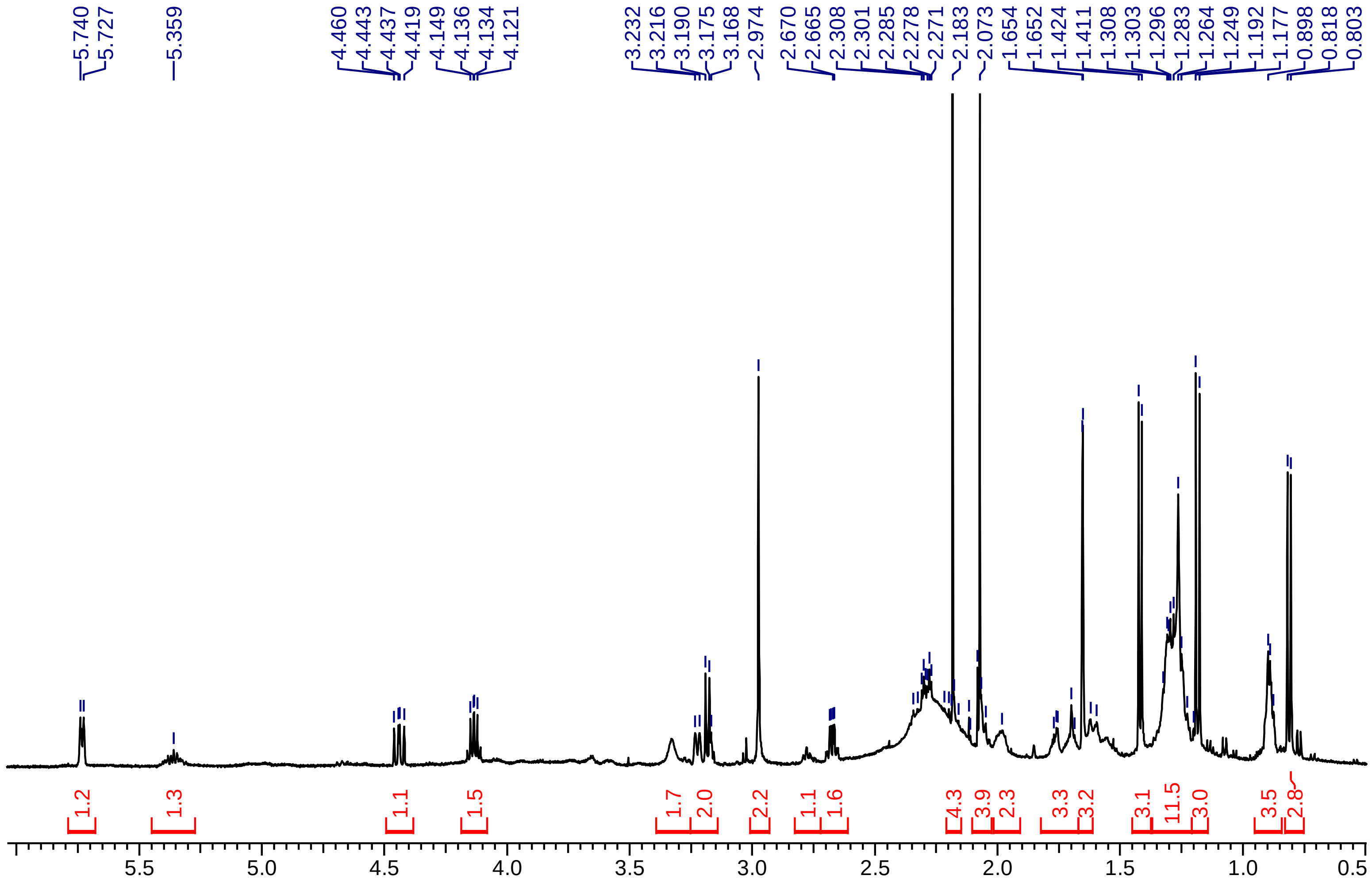

Figure S9: ^{1}H NMR (500 MHz, $CDCl_3$) spectrum of simplicilone B (**2**)

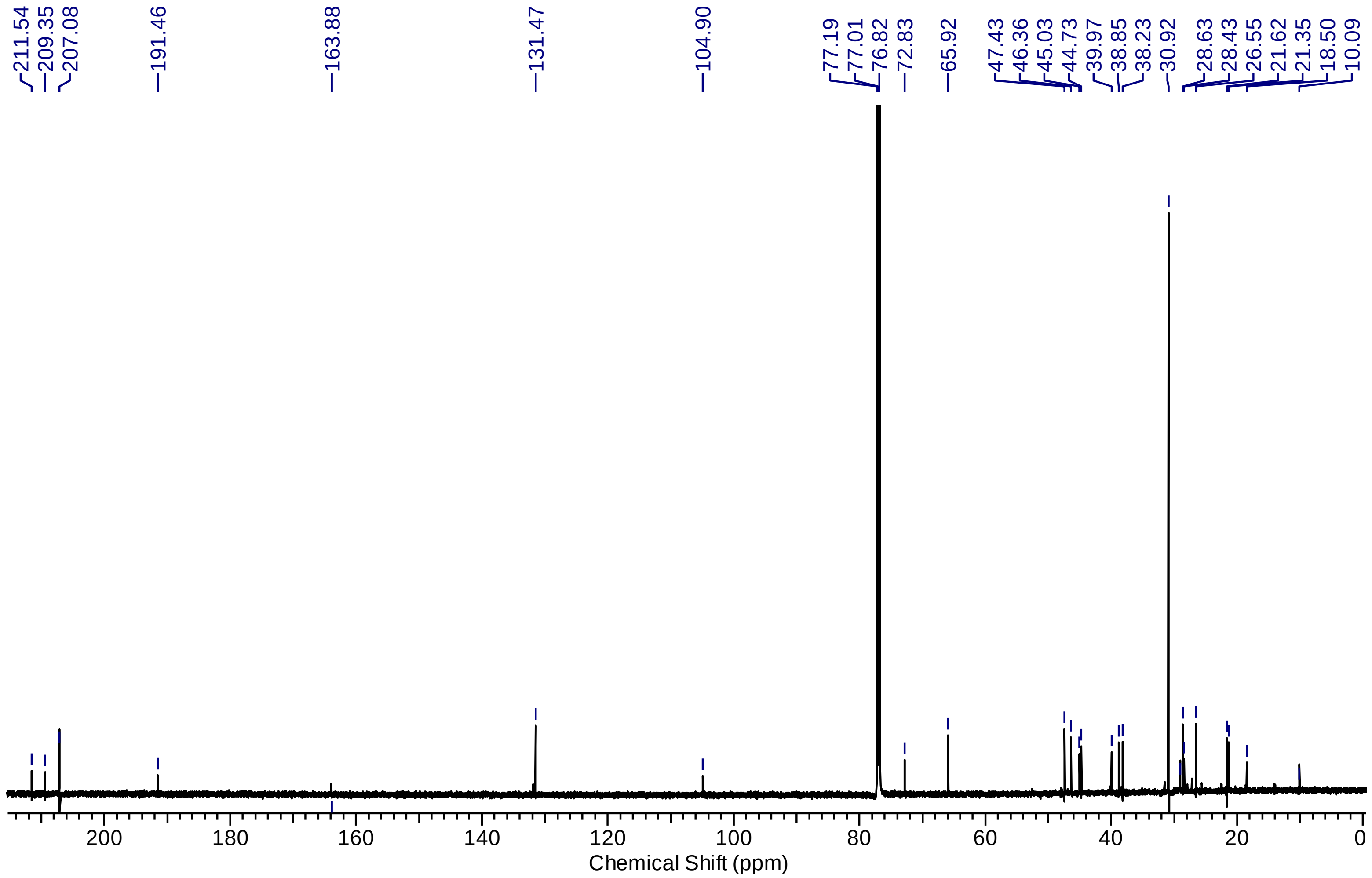

Figure S10: ^{13}C NMR (500 MHz, $CDCl_3$) spectrum of simplicilone B (**2**)

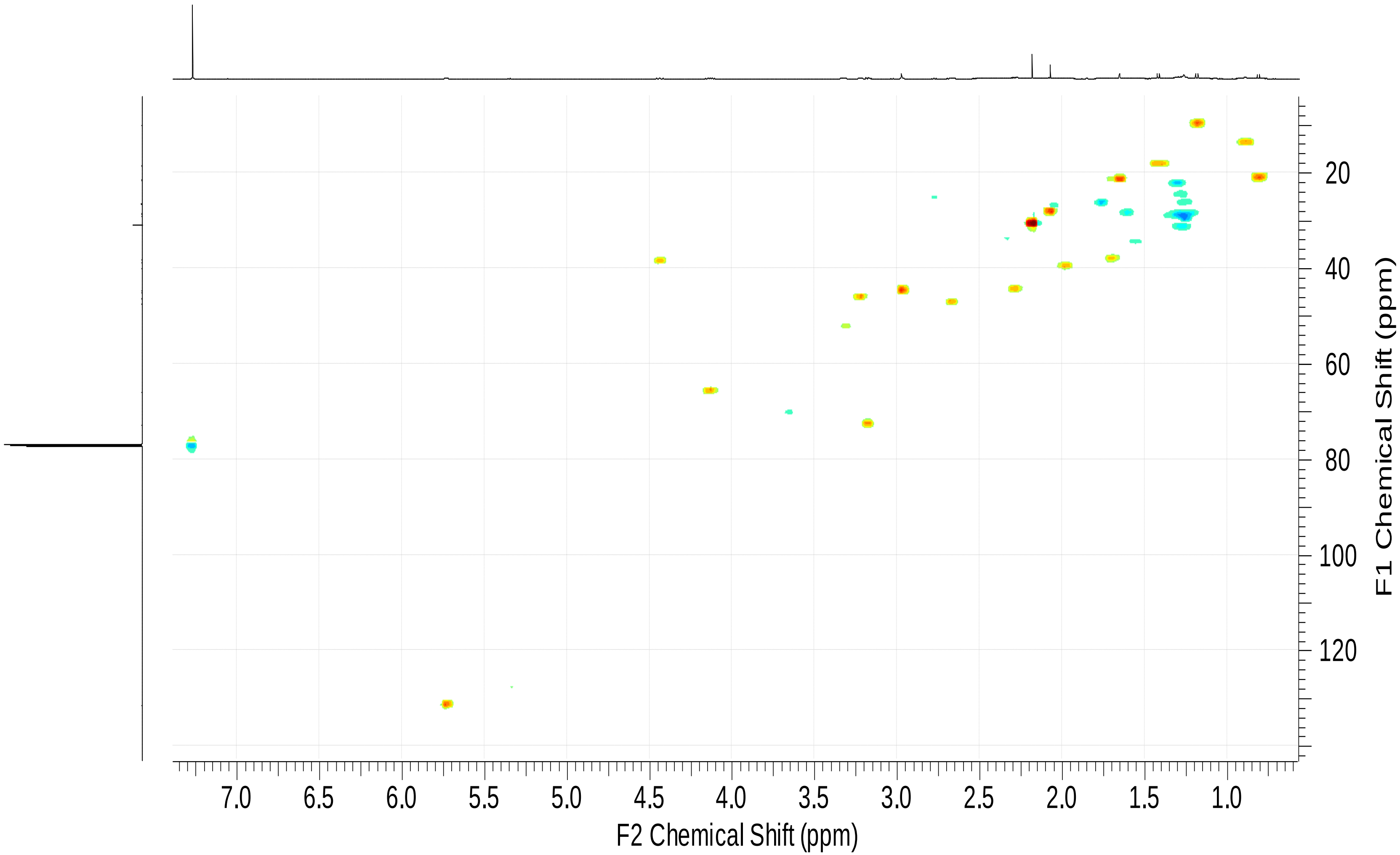

Figure S11: HSQC spectrum of simplicilone B (**2**)

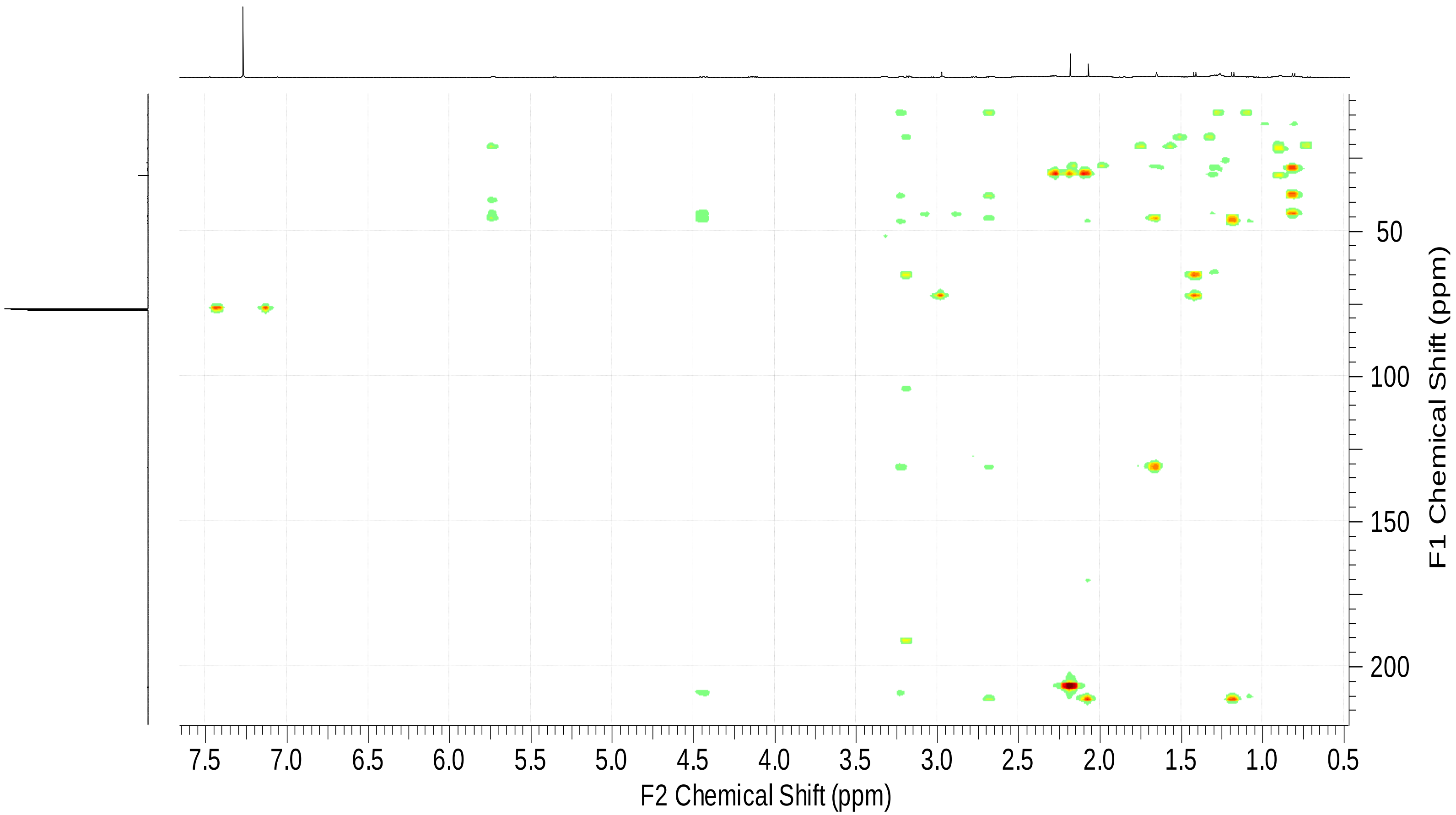

Figure S12: HMBC spectrum of simplicilone B (**2**)

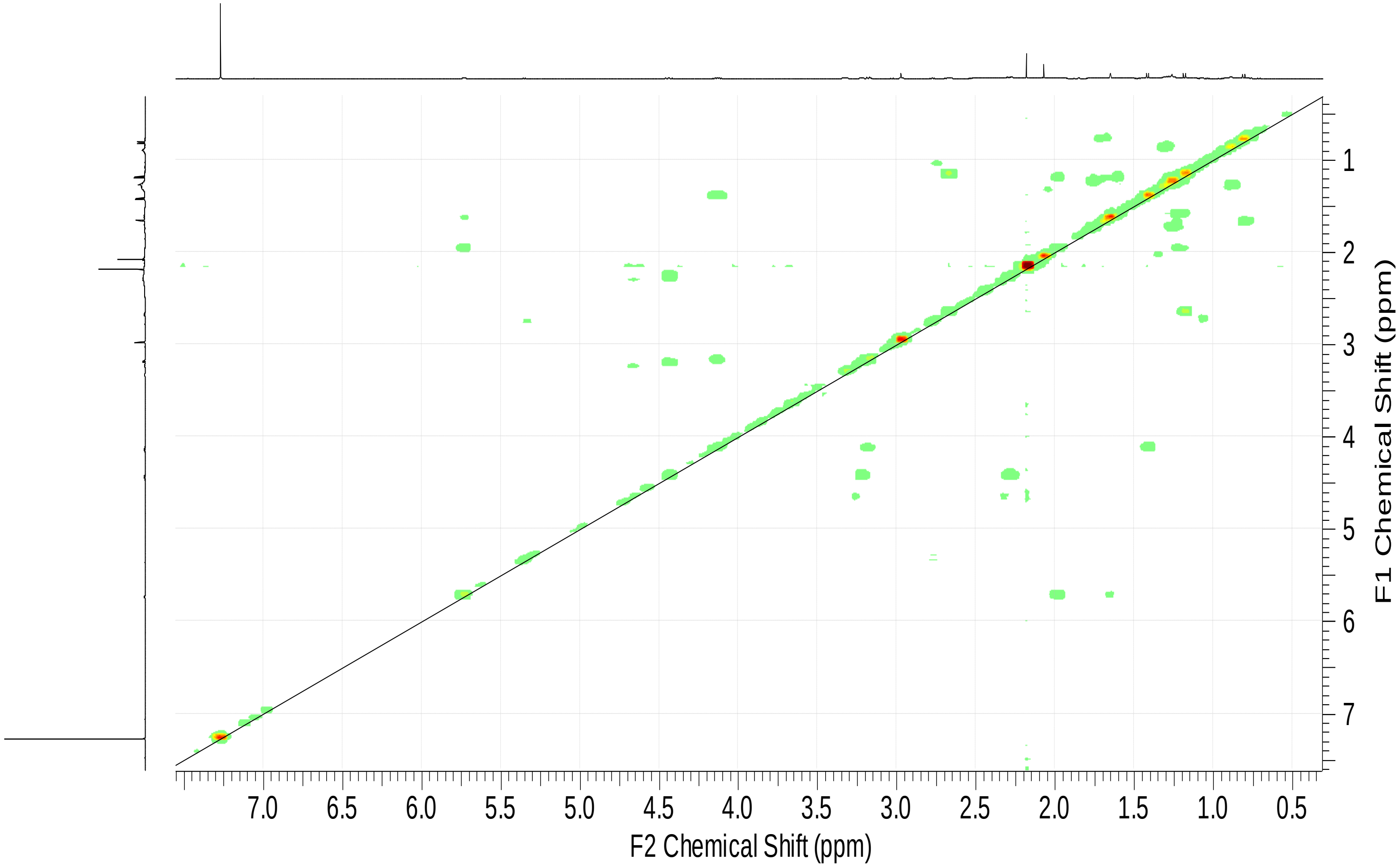

Figure S13: COSY spectrum of simplicilone B (**2**)

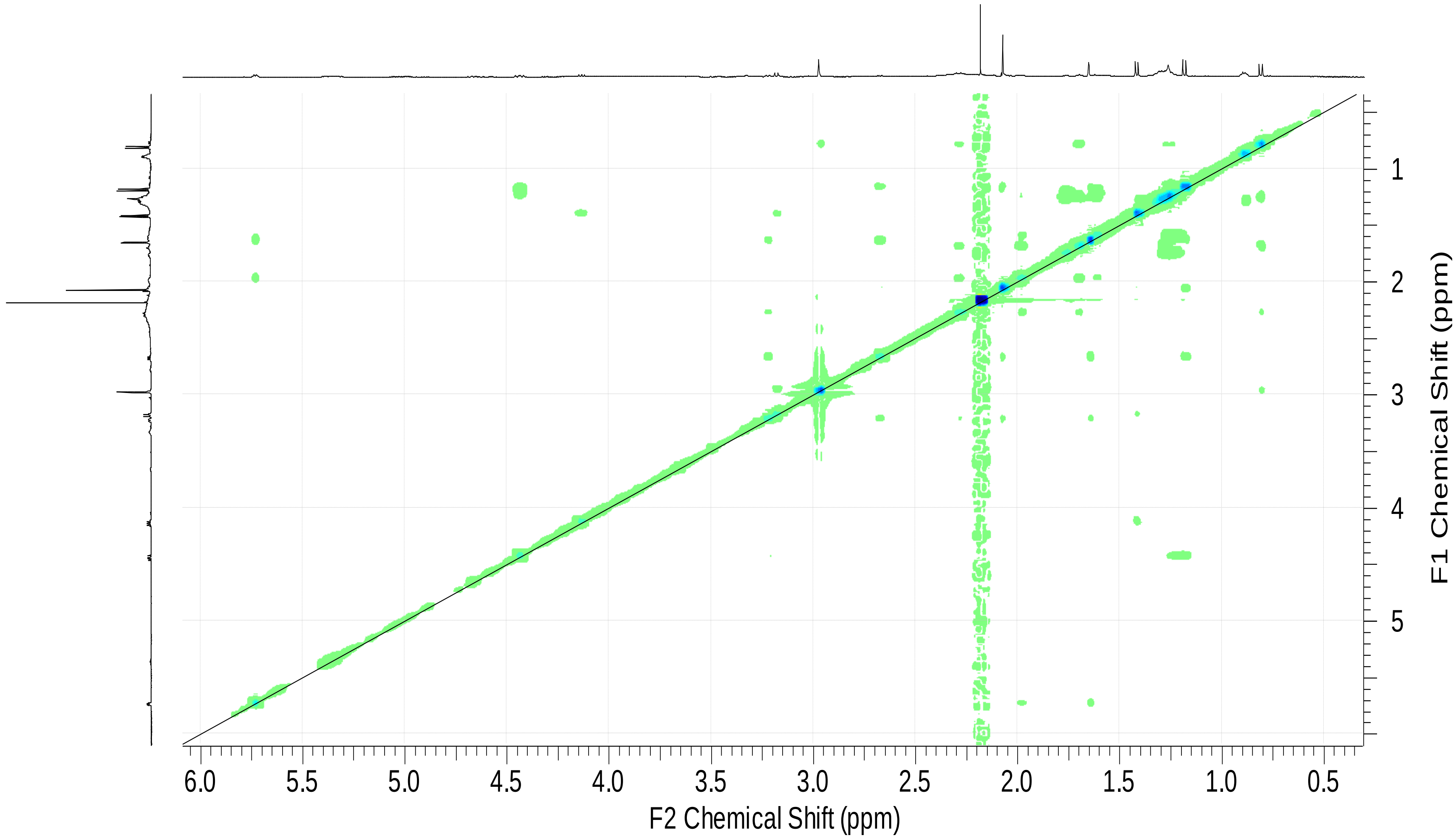

Figure S14: ROESY spectrum of simplicilone B (**2**)

References

Altschul SF, Gish W, Miller W, Myers, EW, Lipman DJ. (1990) Basic local alignment search tool. J. Mol. Biol. 215:403-410.

Gardes M, Bruns TD (1993) ITS primers with enhanced specificity for basidiomycetes – application to the identification of mycorrhizae and rusts. Mol Ecol 2:113–118.

Kearse M, Moir R, Wilson A, Stones-Havas S, Cheung M, Sturrock S, Buxton S, Cooper A, Markowitz S, Duran C, Thierer T, Ashton B, Mentjies P, Drummond A (2012) Geneious basic: an integrated and extendable desktop software platform for the organization and analysis of sequence data. Bioinformatics 28:1647–1649.

Kuhnert E, Fournier J, Peršoh D, Luangsa-ard JJD, Stadler M (2014a) New Hypoxylon species from Martinique and new evidence on the molecular phylogeny of Hypoxylon based on ITS rDNA and β-tubulin data. Fungal Divers. 64:181–203.

White TJ, Bruns TD, Lee S, Taylor JW (1990) Amplification and direct sequencing of fungal ribosomal RNA for phylogenetics. In: Innis MA et al (eds) PCR protocols: a guide to methods and applications. Academic, San Diego, pp. 315–322.
